# 3D Printed Metal Oxide-Polymer Composite Materials for Antifouling Applications

**DOI:** 10.3390/nano12060917

**Published:** 2022-03-10

**Authors:** Andrianna Bouranta, Ioan Valentin Tudose, Luciana Georgescu, Anna Karaiskou, Nikolaos Rafail Vrithias, Zacharias Viskadourakis, George Kenanakis, Efsevia Sfakaki, Nikolaos Mitrizakis, George Strakantounas, Nikolaos Papandroulakis, Cosmin Romanitan, Cristina Pachiu, Oana Tutunaru, Lucian Barbu-Tudoran, Mirela Petruta Suchea, Emmanouel Koudoumas

**Affiliations:** 1 Center of Materials Technology and Photonics, Hellenic Mediterranean University, 71410 Heraklion, Greece; mpandrianna@gmail.com (A.B.); tudose_valentin@yahoo.com (I.V.T.); luciana@hmu.gr (L.G.); ankaraiskou@gmail.com (A.K.); 2 Institute of Electronic Structure and Laser, Foundation for Research & Technology-Hellas, 70013 Heraklion, Greece; nvrithias@materials.uoc.gr (N.R.V.); zach@iesl.forth.gr (Z.V.); gkenanak@iesl.forth.gr (G.K.); 3 Department of Materials Science and Technology, University of Crete, 70013 Heraklion, Greece; 4 Institute of Marine Biology, Biotechnology and Aquaculture, Hellenic Centre for Marine Research, 71500 Heraklion, Greece; efsevia@hcmr.gr (E.S.); nmitrizakis@hcmr.gr (N.M.); georgestr@hcmr.gr (G.S.); npap@hcmr.gr (N.P.); 5 National Institute for Research and Development in Microtechnologies (IMT-Bucharest), 023573 Bucharest, Romania; cosmin.romanitan@imt.ro (C.R.); cristina.pachiu@imt.ro (C.P.); oana.tutunaru@imt.ro (O.T.); 6 Electron Microscopy Center “Prof. C. Craciun”, Faculty of Biology & Geology, “Babes-Bolyai” University, 400006 Cluj-Napoca, Romania; lucian.barbu@ubbcluj.ro; 7 Electron Microscopy Integrated Laboratory, National Institute for R&D of Isotopic and Molecular Technologies, 400293 Cluj-Napoca, Romania

**Keywords:** ZnO based ABS composites, 3D printing, antifouling properties, aquaculture

## Abstract

Current technology to prevent biofouling usually relies on the use of toxic, biocide-containing materials, which can become a serious threat to marine ecosystems, affecting both targeted and nontargeted organisms. Therefore, the development of broad-spectrum, less toxic antifouling materials is a challenge for researchers; such materials would be quite important in applications like aquaculture. In this respect, surface chemistry, physical properties, durability and attachment scheme can play a vital role in the performance of the materials. In this work, acrylonitrile butadiene styrene (ABS)/micro ZnO or nano ZnO composite lattices with different metal oxide contents were developed using 3D printing. Their antifouling behavior was examined with respect to aquaculture applications by monitoring growth on them of the diatoms *Navicula* sp. and the monocellular algae *Chlorella* sp. with image analysis techniques. As shown, the presence of metal oxides in the composite materials can bring about antifouling ability at particular concentrations. The present study showed promising results, but further improvements are needed.

## 1. Introduction

Aquaculture is one of the fastest-growing industries globally. In 2018, world aquaculture fish production reached 82.1 million tons, as well as 32.4 million tons of aquatic algae and 26,000 tons of ornamental seashells and pearls, bringing the total to an all-time high of 114.5 million tones [1]. Because of this increase, aquaculture faces not only the challenge of demand, but also of environmental sustainability [2,3,4,5]. Aquaculture is an important human activity in both economic and nutritional terms.

One problem that aquaculture activities are facing is biofouling, which is the spontaneous colonization by aquatic micro/macro-organisms (freshwater/seawater) on submerged surfaces. This process can rapidly lead to the blocking of fishnet openings, which can restrict the exchange of water with the surrounding environment, and subsequently, give rise to poor water quality and the growth of populations of micro-organisms in fish enclosures. These issues can seriously affect fish populations and lead to increased mortality, increased disease risk and reduced well-being of the fish [6,7,8,9,10,11]. Moreover, biofouling growth can significantly increase the weight of the nets and drag them down, thus affecting the structural integrity of the fish farm and limiting its ability to handle extreme weather conditions [12,13]. Regarding shellfish aquaculture, biofouling results in the reduction of their fitness (i.e., survival, growth, condition and weight); this is directly attributed to competition for food, oxygen and other resources, and indirectly to smothering or interfering with proper valve functioning [14]. Several calcareous fouling species settle on shellfish and affect their aesthetics, often resulting in devalued or discarded products, leading to economic losses [15].

In general, controlling biofouling in fish farming is very challenging and expensive; routine checks and cleaning are essential tasks, as well as the replacement of fish nets and the structural components of the aquaculture system [16]. Biofouling in open sea fish-farming can induce high maintenance costs and reduced return-on-investment, since the preservation of standard operations requires continual control and expensive clean up routines. It is estimated that 5–10% of the production costs in the aquaculture industry are associated with biofouling control [17]. At the same time, frequent fishing net replacement or the use of chemical antifouling agents are stressors for fish. Moreover, the environmental cost of using chemicals is measurable in organisms living around fish farms [18]. 

In general, the technology used at present to prevent biofouling relies on toxic, biocide-containing materials which can become a serious threat to marine ecosystems, affecting both targeted and nontargeted organisms [19]. The development of broad-spectrum, less toxic antifouling materials is a challenge for researchers [20,21].

In this context, one way to introduce antifouling behavior is the addition of antifouling properties to fishnets and fishery-related infrastructure; this is a very important and challenging research topic for scientists working in the materials and nanotechnology fields. The usual ways to prevent biofouling are based on the addition of biocide paints on nets, as discussed in various excellent review papers [22,23].

In recent years, efforts have been made to develop alternative materials for the construction of nets that combine antifouling, durability, ease of production and low cost. These antifouling solutions are usually based on surface-deposited materials, which can nonetheless be released into the environment, leading to undesired environmental pollution and secondary toxic effects for the ecosystem. A recent study [24] demonstrated that ZnO supported nanorods (NRs) have less toxicity compared to other nanostructures in the marine environment. This suggests that ZnO based materials could be used as an environmentally benign alternative to toxic antifouling paints/coatings. Additionally, the photocatalytic activity of ZnO semiconductors may further enhance the antifouling properties, as demonstrated by Sathe et al. in 2017 [25], who employed ZnO nanorod coatings on nylon fishing lines. The antifouling efficiency of the ZnO nanocoating and paint regarding biofilm assemblage was analyzed using metagenomic approaches. Unlike biocide materials, ZnO nanocoating can prevent biofouling by photocatalytic action, representing an environmentally friendly technique. In their paper, Sathe et al. [25] reported the successful development of sunlight-responsive antifouling ZnO nanorod coatings for fishing nets that were more efficient at mitigating biofouling compared to commercial biocidal paints. They noted that this prevention mechanism is an imitation of naturally occurring processes, leading to the selective accumulation of microorganisms which are beneficial to the aquaculture industry and human health. 

Regarding polymer matrices, studies showed that acrylonitrile butadiene styrene (ABS) is a relatively nontoxic material when employed in marine conditions [26,27], i.e., when used in composites with nano- and micro- particulate ZnO, and it may provide a feasible solution for nonpolluting marine antifouling materials, as it was proved that the level of Zn leaching was significant when used in coatings [28].

Based on these findings, the present work is based on embedding ZnO nano- and micro- powders within ABS polymeric matrices, in an effort to produce “fishnet-like” structures with 3D printing that combine germicide and photocatalytic actions. ABS was chosen also for its suitability for 3D printing applications and its good mechanical properties and durability [29]. This approach is novel in that it deals with the fabrication of custom ZnO-ABS composite materials by 3D printing. ZnO-ABS composite materials fabricated by 3D printing have been the subject of a few studies, most of which focused mainly on the mechanical and dielectric properties of such composites [30,31,32,33,34]. Moreover, ZnO-ABS composite filaments are not yet commercially available, and no study to date has reported the fabrication of filaments or the optimization of the 3D printing process with respect to the composition of the material. For these reasons, the first challenge was to fabricate filaments which were suitable for 3D printing, and then to obtain 3D printed materials in the form of grids to be used for marine antifouling applications. In this respect, various ZnO nano- and micro- sized particles–ABS composite filaments were produced and characterized by XRD and SEM in order to observe their homogeneity and uniformity. Then, these filaments were employed to 3D print composite grids that were also characterized by XRD, SEM and Raman spectroscopy, in order to verify the transfer quality of the materials. The composite grids were finally exposed to sea-like conditions in order to investigate their antifouling properties. Finally, all tested materials were characterized by SEM, XRD and Raman spectroscopy after exposure to aquaculture conditions in a trial intended to evaluate both the antifouling ability of the composite materials and the effect of the micro-organisms on the materials themselves.

## 2. Experimental

### 2.1. Materials and Methods

Materials: Acrylonitrile Butadiene Styrene (ABS) from INEOS Styrolution (Frankfurt, Germany). Sigma-Aldrich >97% <50 nm particle size, zinc oxide ZnO nano, 677,450, (Sigma-Aldrich, St. Louis, MO, USA), Sigma-Aldrich >99%, <5 μm average particle size, zinc oxide ZnO micro, 96,479. 

### 2.2. Filament Production

ZnO nano- or micro- powders and ABS pellets were mixed in the proportions presented in Table 1 and forwarded to a “Noztek Pro” (Noztek, Shoreham, West Sussex, UK) high temperature extruder, to be processed at 220 °C, in order to be transformed to cylindrical filaments with a diameter of 1.75 ± 0.15 mm, suitable for 3D-printing. All extrusion parameters, such as extrusion velocity and temperature, were optimized for the production of uniform, continuous cylindrical cords with an overall length of ~ 5 m.

### 2.3. 3D Printing of “Fishnet-like” Grid Structures

**Figure 1:**
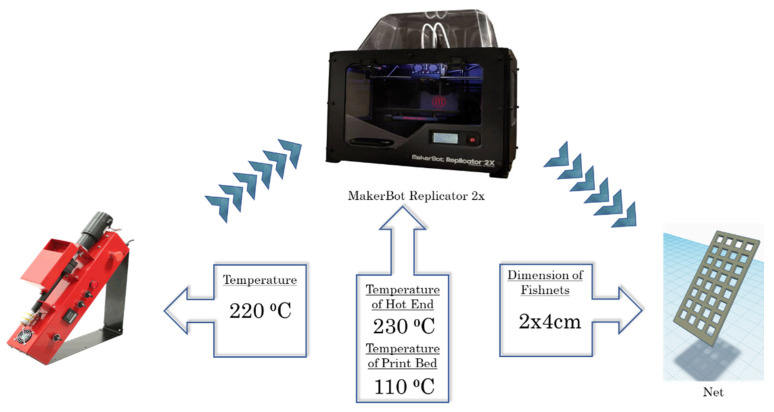
The used printing system and the growth conditions.

Flat, rectangular-shaped “fishnet-like” grid 3D structures (2 cm × 4 cm) were designed using “Tinkercad”, a free online 3D design and 3D printing software from Autodesk Inc. (Mill Valley, CA, USA). A dual-extrusion FDM-type 3D printer (Makerbot Replicator 2X; MakerBot Industries, Brooklyn, NY, USA) was used for the fabrication of the ZnO-ABS nanocomposite samples, using the ZnO-ABS nanocomposite filaments described above. The FMD process of building a solid object involves heating the fed filament and pushing it out layer-by-layer through a heated (230 °C) nozzle (0.4 mm inner diameter) onto a heated surface (110 °C) via a computer controlled three-axis positioning system (with a spatial resolution of approximately 100 μm in the z-axis and 11 μm in x and y). The used printing system and growth conditions are presented in Figure 1.

### 2.4. Characterization Methods

The obtained materials were characterized by SEM, XRD and Raman Spectroscopy, and their antifouling properties were evaluated.

In order to investigate the formation and morphology of the obtained nanocomposite materials, SEM characterization of the nano- and micro-ZnO-ABS filaments, as well as the printed samples, was performed using a Nova NanoSEM 630 (FEI Company, Hillsborough, OR, USA) field emission scanning electron microscope, making it possible to obtain better resolution and gain further insights into their surface structure. All samples were characterized in high vacuum mode without any coating.

SEM characterization of the nano- and micro-ZnO-ABS printed composite materials was also performed after the antifouling experiments using a Hitachi, SU-8230 field emission gun scanning electron microscope (Chiyoda, Tokyo, Japan) equipped with an Oxford Scientific energy-dispersive X-ray spectrometer (Oxford, UK). Samples were sputter-coated with 7 nm Pt/Pd in an Agar Scientific Automated Sputter-Coater (Stansted, Essex, UK). 

X-ray diffraction (XRD) investigations were performed using a Rigaku Ultra high-resolution triple axis multiple reflection SmartLab X-ray Diffraction System (Osaka, Japan) with grazing incidence geometry varying the 2θ from 10 to 60° at a speed of 5°/min. During the measurements, the incidence angle was kept at 0.5°. Peak indexing was achieved using the ICDD (International Center for Diffraction Data) database. 

Micro-Raman spectroscopy was performed at room temperature using a WiTec Raman spectrometer (Alpha-SNOM 300 S, WiTec GmbH, Ulm, Germany) with 532 nm excitation from a diode-pumped, solid-state laser with a maximum power of 100 mW. The incident laser beam was focused to a spot-size of about 1.0 µm on the sample with a 20× working distance microscope objective. The Raman spectra were collected with an integration time 0.043 s and the scattered light was collected by the 20× objective with back-scattering geometry using a 600 grooves/mm grating. Micro-Raman spectroscopy was used to map the sample surface (25 µm × 25 µm), with lateral resolution of up to 0.8 μm. The laser spot scanned the investigated sample area with a preset step size (150 points per line and 150 lines per image), and Raman spectra were acquired pixel by pixel with a scan speed of 6.5 s/line. Spectrometer scanning data collection and processing were carried out using the WiTec Project Five software (CompanyWiTec GmbH, Ulm, Germany). 

**Figure 2:**
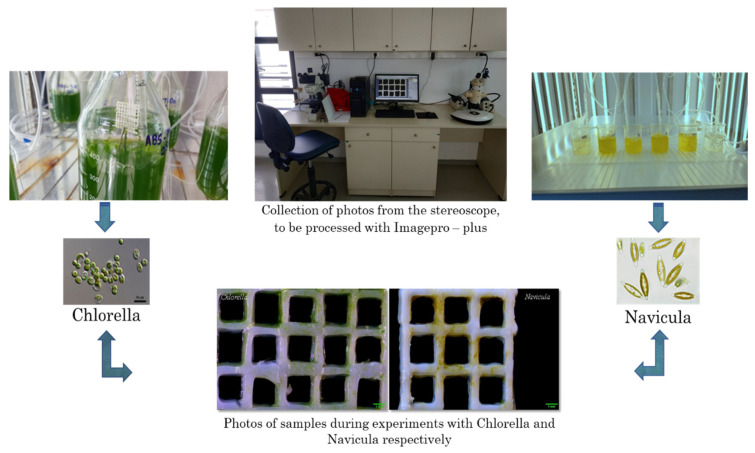
Schematic presentation of experiments for the investigation of the antifouling action.

The antifouling action was investigated by monitoring the growth of the diatoms *Navicula* sp. or monocellular algae *Chlorella* sp. For this investigation, a chamber was employed in which the plankton cultures were maintained under constant conditions (temperature, light, aeration). Samples were placed in the cultures for a specific period of time. The area covered with algae was estimated using image analysis techniques, as schematically presented in Figure 2.

For the algae culture, a standard culture protocol was followed until the desired density was achieved. For *Chlorella*, the initial density was 2.5 M cells mL^−1^, 400 mL of which was placed in a laboratory glass flask. This sample was enriched with fertilizer (Cell-Hi F2P) at a concentration of 0.04 g per bottle. The duration of the experiment was 10 days, with temperature (25 °C) and salinity (25‰) being kept constant. The photoperiod was 24 h daily and the light intensity was 20–50 μmol m^−2^·s^−1^. For *Navicula*, the initial density was 0.307 M cells ml^−1^, of which a volume of 400 mL was placed in a laboratory glass flask. Sodium metasilicate nonahydrate and Cell-Hi F2P were used for lubrication at densities of 0.012 g and 0.04 g per flask, respectively. The temperature was kept constant at 18 °C and the salinity at 30‰ throughout the experiment (10 days). Lighting remained on throughout the twenty-four hours; its intensity ranged from 70 to 120 μmol m^−2^·s^−1^.

In each case, a sample was immersed in the phytoplankton in order to assess the level of adhesion to it. After ten days, the algae density was assessed and the materials were photographed with a stereoscopic camera (Basler, Exton, PA, USA) (Figure 2). The photographs were then analyzed using Imagepro-plus (Meyer instruments, Houston, TX, USA) to estimate the percentage of phytoplankton adherence to the total surface of the material (Figure 2). A total of three groups of experiments were performed using the two different genera of algae with different types of materials.

## 3. Results and Discussion

### 3.1. XRD Characterization

**Figure 3:**
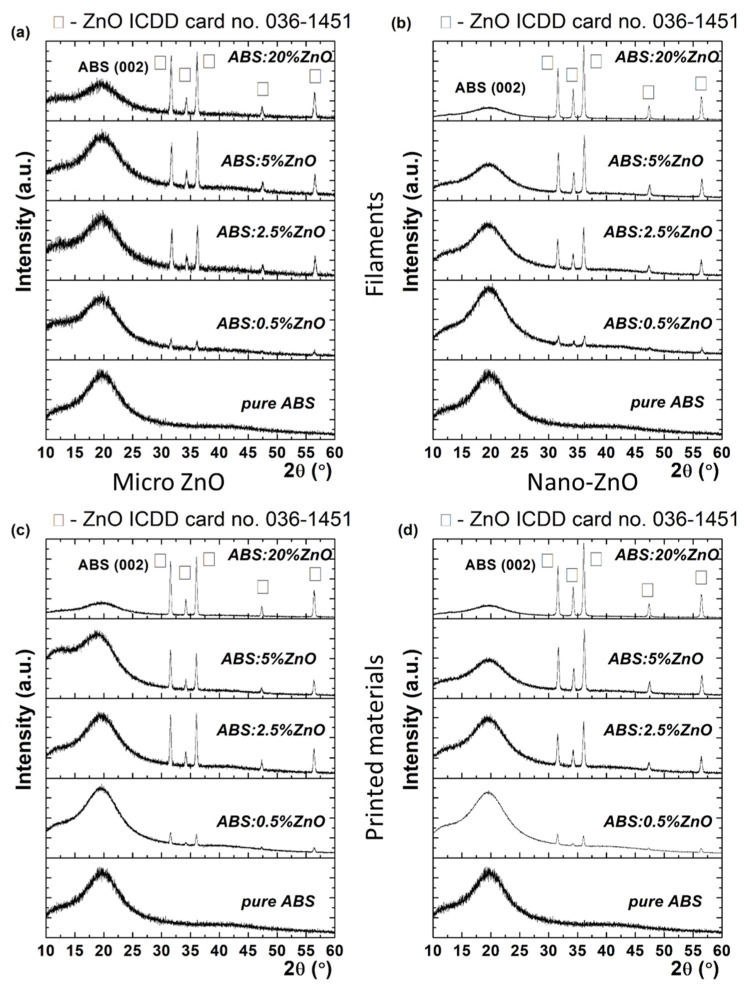
XRD patterns of micro- (**a**) and nano-ZnO-ABS (**b**) filaments, micro- (**c**) and nano-ZnO-ABS (**d**) printed materials, as well as for pure ABS.

Firstly, the properties of the fabricated filaments were studied and their transfer to the printed material was verified. Figure 3a,b shows X-rays diffractograms of the nano- and micro-ZnO-ABS filaments and printed composite materials, respectively.

The XRD profiles exhibited a broad diffraction feature at around 20°, which was due to ABS, accompanied by various diffraction peaks at higher 2θ angles, namely 31.7°, 34.3°m 36.2°, 47.5° and 56.67°. These were unambiguously assigned as different reflections with (100), (002), (101), (102) and (110) Miller indices of wurtzite zinc oxide (w-ZnO) with a = b = 0.32 nm, c = 0.52 nm (P63mc space group), according to ICDD card no. 36-1451. The coexistence of ABS and ZnO phases indicated that the zinc oxide micro-/nano- particles had been successfully embedded in the ABS polymeric matrix. Moreover, since the angular position of the diffraction peaks did not change at different ZnO concentrations, it was concluded that the lattice constant has been preserved by virtue of the Bragg’s law, which expresses the interplanar distance, dhkl, as a function of angular position 2θ, through the equation 2dsinθ= λ, where λ is the X-ray wavelength. In addition, from a visual inspection of diffractograms, as the ZnO concentration increased (e.g., from 0.5% to 20%), the ABS diffraction intensity decreased. (Figure 3). Quantitatively, the relative intensity ratio (RIR) values could be calculated to quantify the ZnO concentration in the ABS matrix. RIR values were calculated considering the integral area of each diffraction peak after fitting with a Voigt profile. In the case of micro-ZnO, the RIR value of ZnO increased from 2.9% (e.g., 0.5% ZnO) to 9.1% (e.g., 2.5% ZnO), then to 17.9% (e.g., 5% ZnO), reaching a value of 33% (e.g., 20% ZnO). In the case of nano-ZnO, the crystallization process seemed different, since the RIR values were different, reaching 46% at the highest ZnO concentration (20%). The different crystallization phenomena could also be attributed to the different sizes of the crystalline domains, τ, as proved by the Scherrer equation [35], which relates the Full Width at Half Maximum (FWHM) β with the crystal quality, τ = kλβcosθ, where k is the shape, taken as 0.9 in the present study. For instance, the FWHM varied from 0.35° to 0.32° for ZnO micro, while for ZnO nano, a variation from 0.32° to 0.27° was observed. Accordingly, the mean crystallite size in the first case varied between 24 and 26 nm, when the ZnO micro concentration increased from 0.5% to 20%. At the same time, the mean crystallite size increased from 26 nm to 31 nm in the case of ZnO nano. Further, it was observed that the printed materials preserved the unit cell parameters of ZnO in the filaments (i.e., encoded in the peaks position), as well the preferential orientation along (101), as shown in Figure 3c,d. Regarding the RIR value, it was observed that a higher ZnO micro in ABS matrix was obtained, since the RIR value reached 43.5%. According to the Scherrer’s equation, an improved RIR value implies a better crystal quality over the filament composite, reflected in a mean crystallite size of 30 nm. In the case of ZnO nano, the crystal quality of the composite material did not improve in the case of the printed material, even if the RIR value reached 56%. At this point, it was clear that the ZnO concentration induced different crystallization degrees in the composites, as reflected in the size of the crystalline domains, also called the mean crystallite size and the RIR value, that encodes the ZnO percentage in the material. As will be shown in the following section, the ZnO concentration was found to be related also to different surface morphologies. 

### 3.2. SEM Characterization

**Figure 4:**
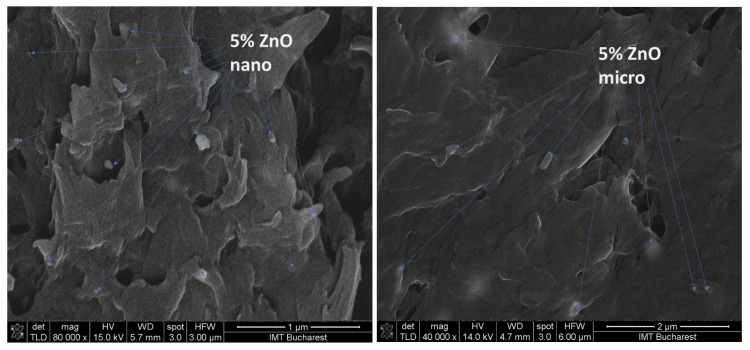
High magnification FESEM for the 5% ZnO nano- and micro- samples; 5% nano ZnO-ABS × 80,000; 5% micro ZnO-ABS × 40,000.

SEM characterization of the nano- and micro-ZnO-ABS filaments and printed composite materials revealed that, in both cases, the filament materials had a noncompact, fluffy architecture, while the 3D printed composites showed a more compact, homogeneous surface. As the ZnO concentration increased, the transformation of the filament material to the 3D object enhanced the homogeneity of the material. Regarding the presence of ZnO in the composite, this was investigated by FESEM at high magnification in situ. A reasonably homogeneous distribution was observed within the ABS matrices in all of the developed composites, as also confirmed by the Raman microscopy. An example of high magnification FESEM for the 5% ZnO nano- and micro- samples is presented in Figure 4.

**Figure 5:**
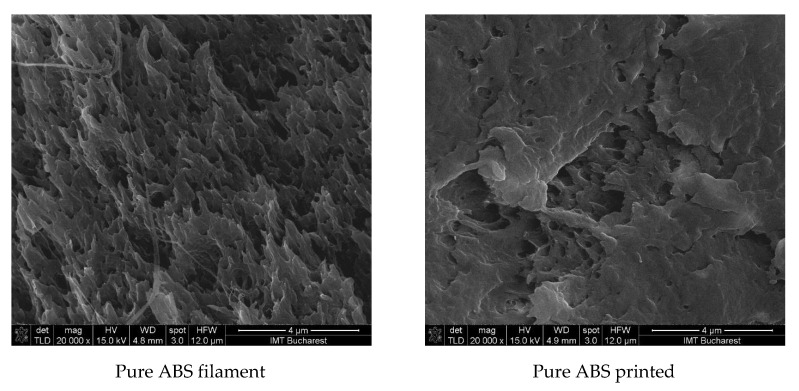

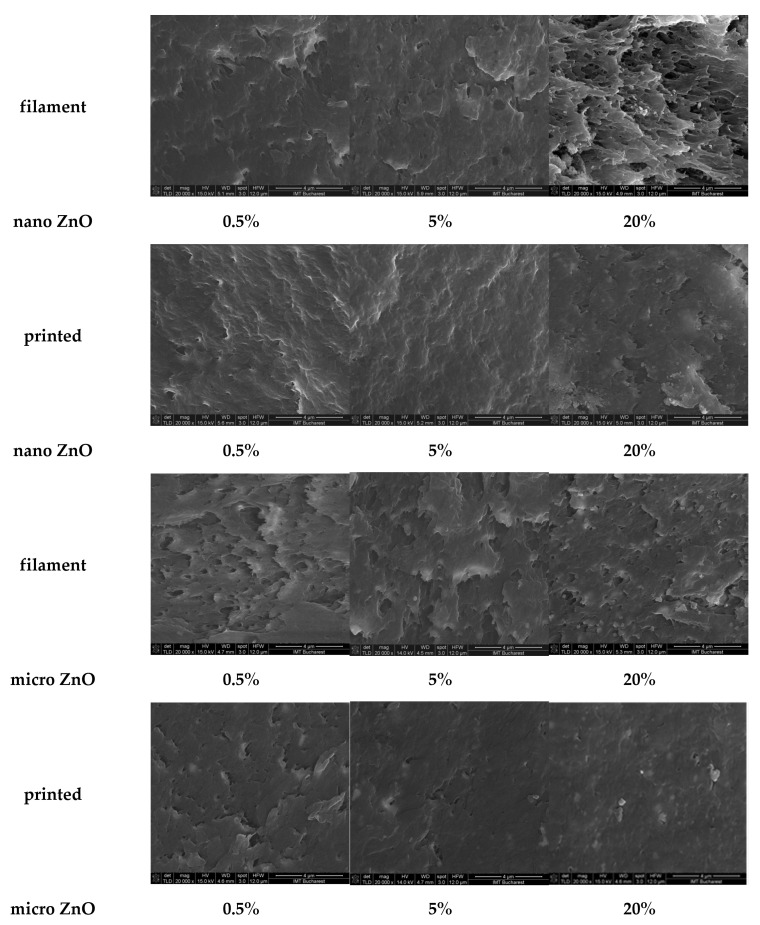
SEM images presenting examples of the structures of the 0.5%, 5% and 20% nano- and micro-ZnO-ABS filaments and printed composite materials. Magnification ×20,000. Scale is 4 µm for all images.

Some examples of the structure of the pure ABS and the 0.5%, 5% and 20% nano- and micro-ZnO-ABS filaments and printed composite materials are shown in Figure 5, where, ZnO particles are also clearly visible onto the surface; in these images, a 20% ZnO particle concentration was used.

The different structuring of the materials in the filaments may have been due to the extrusion process that consisted of pushing the desired mixture in a melted form through an extruder to create the correct diameter filament. To obtain the printed material, the filament was melted again and extruded through a nozzle, usually much smaller than the initial diameter of the filament. To our knowledge, no studies to date have presented a comparison of how the structural and morphological properties of materials, i.e., the shapes of the filaments, change after 3D FDM printing. Since this seems to be the first reported observation of significant structural and morphological property changes of filament materials with respect to their printed versions, further studies regarding this subject are ongoing in order to quantify these observations. 

### 3.3. Raman Characterization

Micro-Raman mapping is known as a very effective tool for the characterization of the structural and chemical homogeneity of small-area, thin deposited films. This technique also yields statistics regarding the distribution of a printed material using the Raman bands present in one mapping. 

Raman mapping and spectroscopy characterization of the nano- and micro-ZnO-ABS printed composite materials revealed the distribution of the filler in the ABS polymeric matrices as its concentration increased. The Raman spectra were then discriminated from each other by chemometric analysis, and the end result was a false color map, i.e., an image of the sample that contained highly precise structural and chemical information.

**Figure 6:**
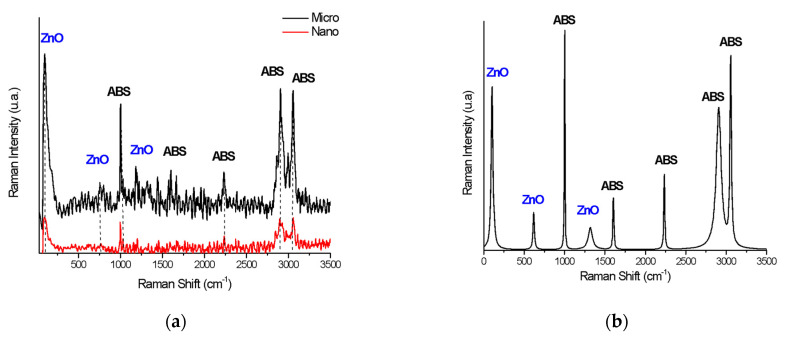

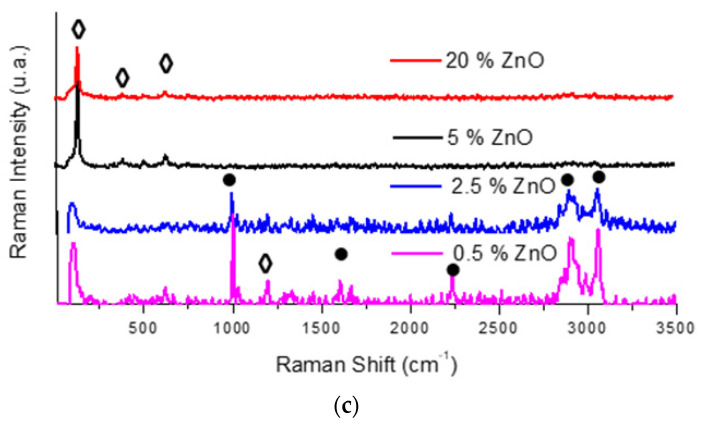
(**a**) Raman spectra of 20% micro- and nano-ZnO-ABS printed composite materials; (**b**) Peak analysis of Raman spectrum for 20% micro-ZnO-ABS; and (**c**) Raman spectrum for micro-ZnO-ABS composites as a function of ZnO concentration.

For this purpose, at least 50 Raman spectra were initially collected from different surface areas of each sample and compared. Figure 6 shows the Raman spectra of the 20% micro- and nano-ZnO-ABS printed composite materials (A) and a peak analysis of the Raman spectrum for 20% micro-ZnO-ABS (B). As one can observe, both the nano- and micro-ZnO containing composites showed characteristic peaks of the constituents but, for the nano-ZnO, the corresponding peaks were very weak. Figure 6 C shows some representative examples of Raman spectra for the micro-ZnO-ABS printed composite materials with various ZnO concentrations. Various peaks related to ABS were observed, e.g., at 1000 cm^−1^, δ (C=C-H) stretching, 1600 cm^−1^, C=C stretch, 2300 cm^−1^ and 2900 cm^−1^ ν_as_ (CH_2_) and ν_s_ (CH_2_) bonds, 3000 cm^−1^, C=C-H styrene aromatic bending [36]. Moreover, peaks related to ZnO were observed at 100 cm^−1^, corresponding to E_2_(low) symmetry mode, 437 cm^−1^, the peak of E_2_(high) mode and 643 cm^−1^, which was related to the simultaneous excitation of LA + LO phonons [37]. As the ZnO microconcentration increased, the representative peaks for ABS decreased in intensity. Similarly, at low frequencies, the specific ZnO peaks showed a slight increase in intensity. For nano-ZnO-ABS samples, due to the relatively homogeneous distribution of the ZnO nanomaterial in the ABS matrix, the intensity of the peaks of the materials did not show a significant change.

Next, 10 different maps were collected in various surface areas of each sample and compared in order to ensure that the material distribution in the sample did not exhibit significant spatial variations.

**Figure 7:**
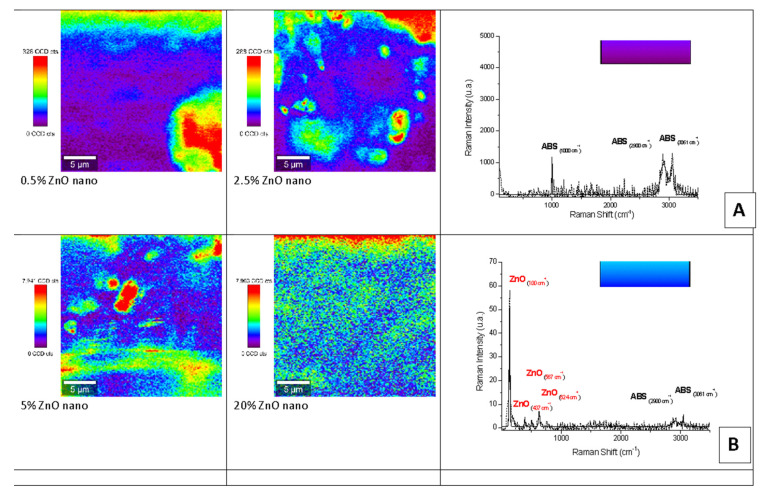
Raman mapping and spectra of nano-ZnO-ABS printed composite materials. (**A**) Example of Raman spectrum corresponding to mauve-blue dark color regions in the maps; (**B**) example of a Raman spectrum corresponding to blue color regions in the maps.

**Figure 8:**
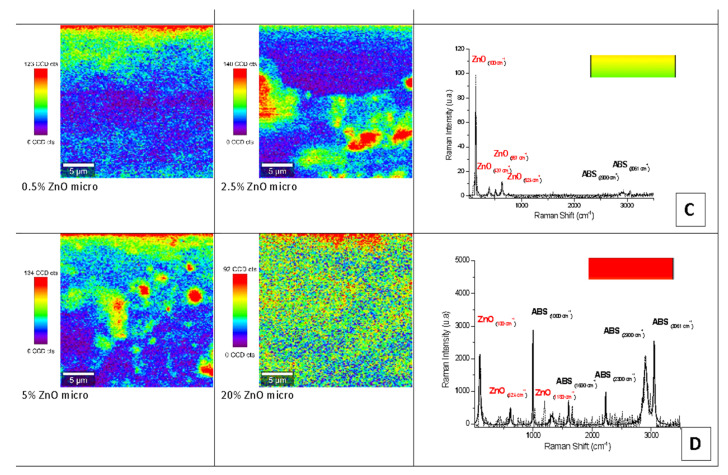
Raman mapping and spectra of micro-ZnO-ABS printed composite materials. (**C**) Example of a Raman spectrum corresponding to yellow-green color regions in the maps; (**D**) example of Raman spectrum corresponding to red color regions in the maps.

Figure 7 and Figure 8 show representative examples of Raman mapping and related spectra for nano- and micro-ZnO-ABS printed composite materials of various concentrations.

Some examples of micro-Raman maps of the nano- and micro-ZnO-ABS composite samples are shown in Figure 7 and Figure 8 respectively. The mauve-blue dark areas in the image correspond to the distribution of the ABS bands. The Raman spectrum of ABS (Acrylonitrile Butadiene Styrene), as shown in Figure 6A for the nano-ZnO-ABS, was characterized by seven main bands located at about 1000 cm^−1^, 1300 cm^−1^, 1439 cm^−1^, 1600 cm^−1^, 2300 cm^−1^, 2900 cm^−1^ and 3061 cm^−1^, which were attributed as follows: the band at 3000 cm^−1^ to aromatic ν (C=C-H) stretching and 1600 cm^−1^ to ν (C=C), at 2900 cm^−1^ and 2300 cm^−1^ to the ν_as_ (CH_2_) and ν_s_ (CH_2_) bonds and the region of 1000–1500 cm^−1^ to the aromatic skeleton stretching γ(CH_2_), as shown in Figure 6 A for the nano ZnO-ABS and 7C for the micro ZnO-ABS. The band at 1000 cm^−1^ was related to δ (C=C-H) stretching. For the case of ZnO, the bands shown as yellow-red areas in the Raman maps comprised a peak at 100 cm^−1^, which was related to the E_2_(low) symmetry mode and the peak of E2 mode at 437 cm^−1^. As shown, as the ZnO concentration increased, the distribution on the surface improved in both samples, reaching uniform coverage, especially in the micro-ZnO containing samples.

### 3.4. Antifouling Properties

**Figure 9:**
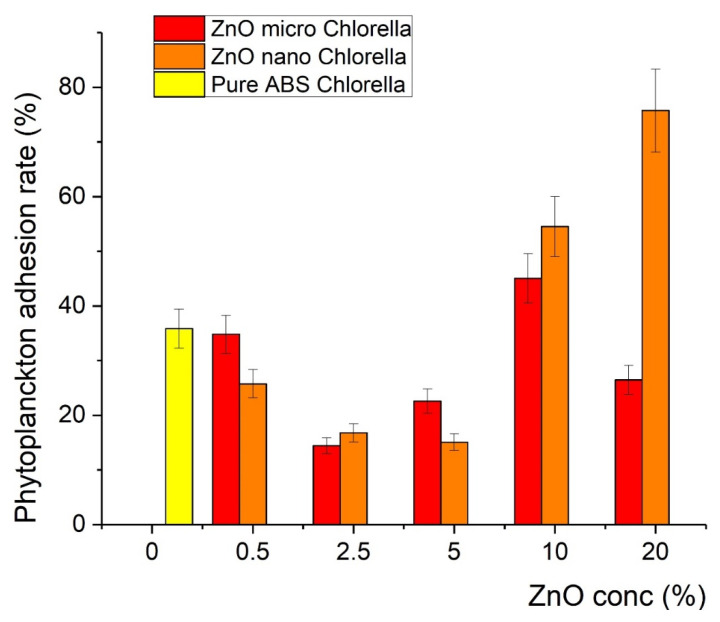
Percentage of coverage of composite material with *Chlorella*.

**Figure 10:**
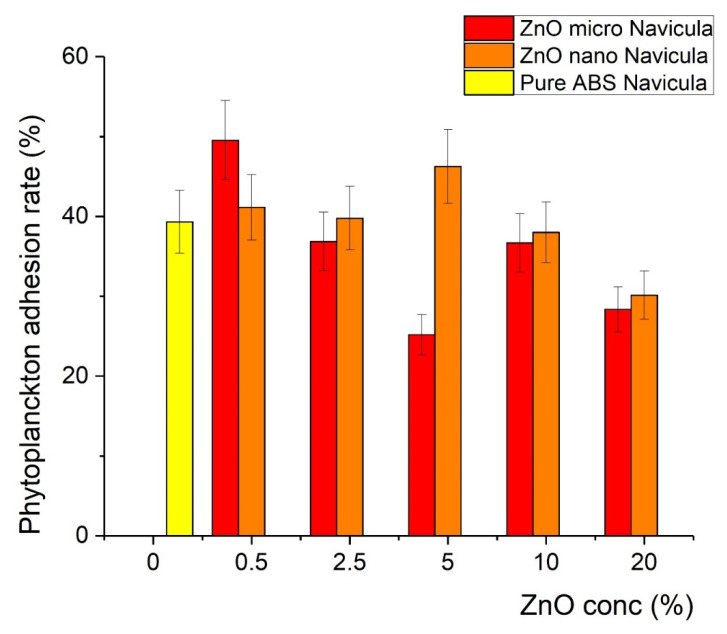
Percentage of coverage of composite material with *Navicula*.

The printed samples were exposed to plankton cultures as described in the Experimental section. It was found that after the incorporation of ZnO in the polymer matrix, the covering of the samples with *Chlorella* and *Navicula* was less effective in particular compositions, indicating antifouling action. In particular, for the *Chlorella* tests, the covering was minimized at a concentration of 2.5% of microsized ZnO (12% coverage), while for the 5% nanosized sample, 15% coverage was recorded, as shown in Figure 8. In contrast, the antifouling action was found to be less effective with *Navicula*, since the minimum coverage achieved was 25% when micro-ZnO was used (Figure 9). Detailed results regarding the effects of ZnO type and concentration on the covering of the samples with *Chlorella* and *Navicula* are presented in Figure 9 and Figure 10.

### 3.5. Properties of the Materials Following Exposure to Plankton

**Figure 11:**
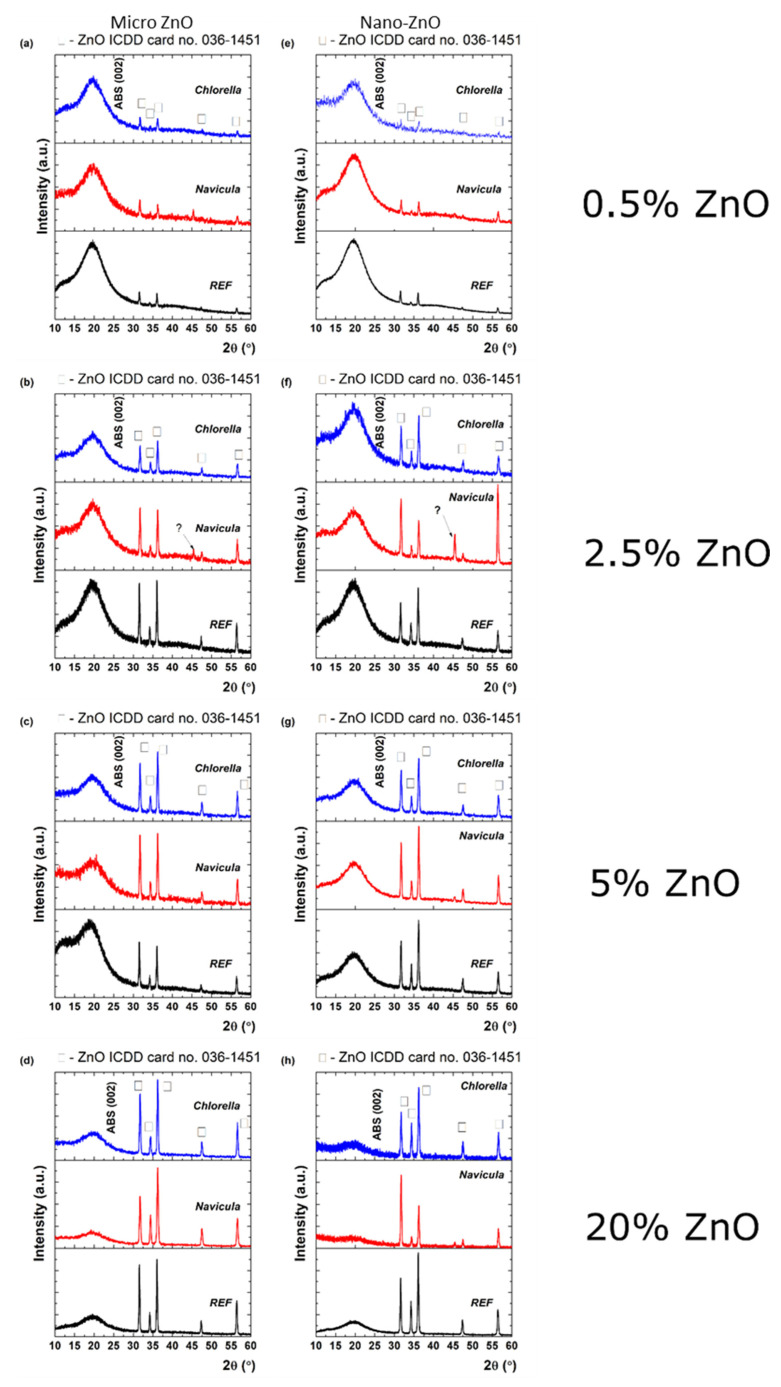
XRD patterns of micro- (**a**–**d**) and nano-ZnO-ABS (**e**–**h**) for the initial printed 3D grid (black) exposed to *Navicula* (red) and *Chlorella* (blue), respectively.

XRD characterization of the materials after antifouling testing showed that the growth of the plankton can influence their properties, as presented in Figure 11. The influence of *Navicula* (red line) and *Chlorella* (blue line) on the initial nano- and micro-ZnO-ABS printed materials (denoted as REF) was studied by means of grazing incidence X-ray diffraction. Although the relative intensity of the ZnO diffraction peaks over the ABS changed after the exposure to *Navicula* and *Chlorella*, the ZnO lattice constant was preserved, as shown by applying Scherrer’s equation in each case to the (100) diffraction peaks. In the case of ZnO micro, the following observations can be made regarding the transition from the initial sample (REF) to the *Navicula* or the *Chlorella* covering: (i) At 0.5% concentration, the FWHM increased from 0.262° to 0.279° and then to 0.312°; this was ascribed to a decrease in the mean crystallite size from 31.8 nm to 26.1 nm. (ii) At 2.5% concentration, the FWHM had values of 0.283°, 0.342°, 0.334°, which implies a decrease of the mean crystallite size from 29.5 nm to 24.4 nm. (iii) At 5% concentration, the FWHM increased from 0.265° to 0.328° and then to 0.337°; this was related to a decrease of the mean crystallite size from 31.5 nm to 25.4 nm, reaching 24.7 nm. (iv) At 20% concentration, the FWHMs were 0.260°, 0.355° and 0.319°, implying mean crystallite sizes of 32.1 nm, 23.5 nm and 26.1 nm. Meanwhile, for nano-ZnO: (i) At 0.5% concentration, the FWHM increased from 0.258° to 0.311° and then 0.397°, which implies a decrease of the mean crystallite size from 32.3 nm to 26.8 nm, reaching 21.0 nm. (ii) At 2.5% concentration, the FWHM presented a variation from 26.5 nm to 24.8 nm and then to 24.3 nm. (iii) At 5% concentration, the mean crystallite size presented variation from 28.6 nm to 27.8 nm and then 23.8 nm. (iv) At 20% concentration, the mean crystallite size varied from 28.6 nm to 26.5 nm and then 24.5 nm. The mean crystallite size values for ZnO before and after exposure with *Navicula* and *Chlorella* are summarized below in Table 2. 

The crystal quality of ZnO was affected after *Navicula* and *Chlorella* exposure in each case. This may have been associated with either a decrease in the ZnO quantity or some shielding effect caused by the biofilm. It also should be noted that exposure to *Navicula* led to the occurrence of an unidentified diffraction peak at 45.3°, the origin of which could not be clearly determined. In the case of 2.5% nano-ZnO-ABS (Figure 11f), even the preferential crystalline orientation of ZnO was modified from (101) to (110). As observed also by EDX mapping analysis, new elements were present in the materials that may have come from the plankton; however, no XRD and EDX characterization references for plankton were found in the literature. In any case, further studies are required and are ongoing to better understand these effects. It is worth mentioning that, to our knowledge, such studies have never been reported in the literature. As such, at this point, no references regarding this specific subject can be considered.

**Figure 12:**
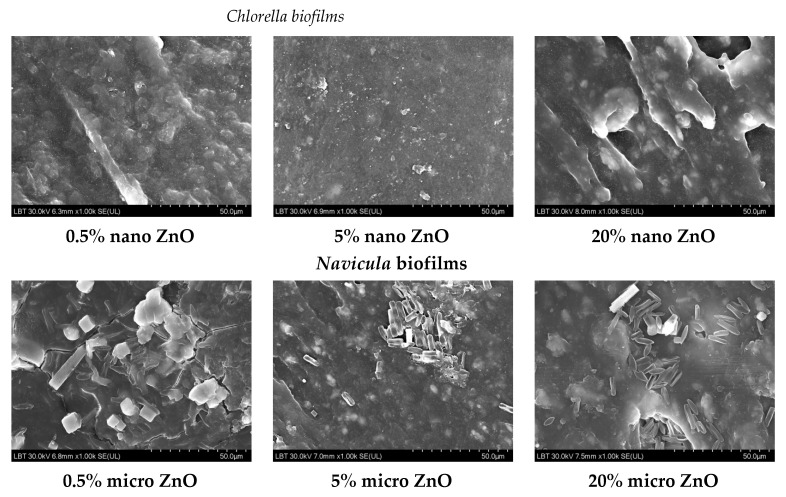
SEM images of *Chlorella* and *Navicula* biofilms on the 0.5%, 5% and 20% nano- and micro-ZnO-ABS 3D printed composites, respectively.

**Figure 13:**
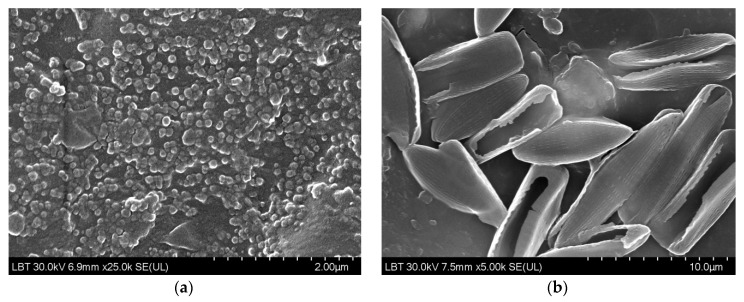
Higher magnification SEM images of (**a**) *Chlorella* biofilm on the 5% nano-ZnO-ABS and (**b**) *Navicula* biofilms on the 5% micro-ZnO-ABS, 3D printed composites.

SEM images of *Chlorella* and *Navicula* biofilms on the 3D printed “fishnet-like” grids were recorded. As shown, their morphology depended on the ZnO concentration, but not really on its size. Based on this observation, Figure 12 presents images of *Chlorella* biofilms on the nano-ZnO containing samples and *Navicula* biofilms on the micro-ZnO ones. As the ZnO concentration increased, the biofilm surfaces seemed to become rougher, suggesting a higher abundance of biomaterials on the surfaces. The smoothest *Chlorella* biofilms were observed on the 5% nano-ZnO containing samples. For the *Navicula* biofilms, notwithstanding the presence of the diatoms, some cubic large crystals were observed, which, based on their appearance, could have been crystallized NaCl salt. This may also have been the cause of the unidentified XRD peak observed in some *Navicula* exposed samples. These can be clearly observed in Figure 13 in the case of 0.5% micro-ZnO and in smaller quantities in the presented larger concentration samples. The smoothest *Navicula* biofilms were observed on the 5% micro-ZnO containing samples, a fact that confirms observations from previous antifouling studies. 

**Figure 14:**
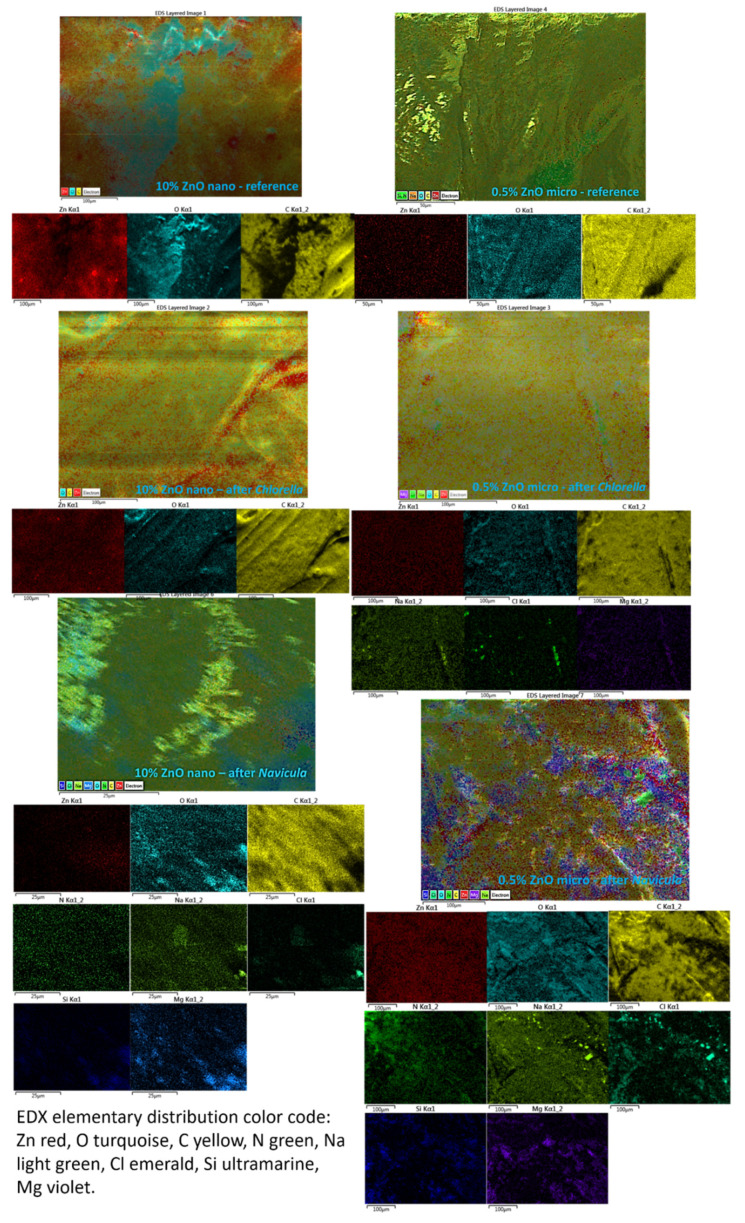
EDX elemental mapping of 10% nano-ZnO-ABS and 0.5% micro-ZnO-ABS, 3D printed composites (reference and after plankton exposure). Large image-composite elemental map followed by individual element mapping bellow.

EDX elemental analysis and mapping of materials exposed to *Chlorella* and *Navicula* were performed; however, due to the instability of ABS to the high energy electron beam in a vacuum, quantitative evaluations were not possible. Some qualitative elemental mapping of the various elements onto 3D composite grid surfaces are presented in Figure 14. As one can observe from Figure 14, the ZnO was uniformly distributed in the material. After exposure to *Chlorella* and *Navicula*, the presence of Na, Cl and Mg was observed. After *Navicula* exposure, Si was also present. Na, Cl and Mg are usually present in seawater while Si was attributed to *Navicula*, as it is a diatom that is rich in SiO_2_.

Regarding previously reported ZnO results regarding antifouling applications, there are only a few papers in the literature related to the use of ZnO incorporated in a polymeric matrix, these concerning usually biofouling of membranes and ZnO is employed in combination with other known antifouling agents such as Cu, or Ag and graphene or graphene oxide. [38,39,40,41,42]. Thanks to the outstanding properties of ZnO nanoparticles, the present researchers were able to improve the antifouling ability by combining ZnO nanoparticles with a polymeric membrane [43,44,45,46,47,48,49,50,51]. Moreover, since ZnO nanoparticles possess a strong hydrophilicity by adhering the hydrophilic functional groups such as –OH, –SO_3_H, and –COOH [52], this can give rise to new approaches to improving antifouling ability. For marine applications, ZnO based coatings have mostly been employed as paints [53,54]. These coating were proved to be quite effective after use in a marine environment for 30 days, according to a report by H.E. Yong, et al. [54]. Those authors prepared a biocide-free antifouling paint using ~30 nm ZnO nanoparticles and an alkyd resin binder with nontoxic additives. This work suggested that the ZnO nanopaint could open new horizons in maritime industries. Another laboratory scale experimental work performed by Marwan Al-Fori et al. [55] reported the antifouling properties of ZnO nanorod coatings that were investigated using the marine bacterium *Acinetobacter* sp. AZ4C, larvae of the bryozoan *Bugula neritina* and the microalga *Tetraselmis* sp. ZnO nanorod coatings were fabricated on microscope glass substrates by a hydrothermal technique using two different molar concentrations (5 and 10 mM) of zinc precursors. These coatings were tested for 5 h under artificial sunlight (1060 W m^−2^ or 530 W m^−2^) and in the dark (no irradiation). In the presence of light, both the ZnO nanorod coatings significantly reduced the density of *Acinetobacter* sp. AZ4C and *Tetraselmis* sp. in comparison to the control (clean microscope glass substrate). High mortality and low settlement of *B. neritina* larvae was observed on the ZnO nanorod coatings subjected to light irradiation. In darkness, neither mortality nor enhanced settlement of larvae was observed. The larvae of *B. neritina* were not affected by Zn^2+^ ions. It was concluded that the ZnO nanorod coatings effectively prevented marine micro- and macro- fouling under static conditions. According to a work by LailaAl-Naamani et al. [56], chitosan-ZnO nanoparticle coatings showed antifouling activity against *Navicula* sp. and antibacterial activity against the marine bacterium *Pseudoalteromonas nigrifaciens*. Additional antifouling properties of the coatings were investigated in a study using tanks containing natural sea water under controlled laboratory conditions. Weekly, for four weeks, biofilm were removed and analyzed by flow cytometry to estimate total bacterial densities on the coated substrates. Chitosan-ZnO hybrid coatings led to better inhibition of bacterial growth in comparison to chitosan coatings alone, as determined by flow cytometry. To date, no other study exists on the potential use of ZnO-ABS materials for marine antifouling applications.

## 4. Conclusions and Perspectives

ABS/micro- and nano-ZnO composite lattices with different ZnO contents were developed using 3D printing and their antifouling behavior was examined with respect to aquaculture applications. The basic characteristics of the composite materials were studied using X-ray diffraction, electron microscopy and Raman spectroscopy before and after 3D printing. Regarding their antifouling action, this was investigated by monitoring the growth on them of the diatoms *Navicula* sp. and the monocellular algae *Chlorella* sp. The areas covered with algae, which indicated the extent of the antifouling capability, were then estimated by applying image analysis techniques. As shown, the ZnO concentration in the composite materials correlated with antifouling ability, as well as the size of the ZnO powders. In addition, the interaction of the samples with *Navicula* sp. and *Chlorella* sp. during the antifouling tests was found to influence their basic characteristics. Regarding the observation of the different effects of ZnO size and concentration on the growth of *Chlorella* and *Navicula*, it seems that the two microorganisms exhibited different growth patterns on the composite materials. Moreover, the optimal ZnO concentration that promoted antifouling seemed to also be connected to the biology of the aforementioned microorganisms. Further studies are ongoing, as these are required before valid conclusions can be drawn regarding the antifouling action of the described materials.

This report, the first to use 3D printing as a fabrication method of “fishnet-like” ZnO-ABS composite materials with enhanced antifouling properties, showed very promising results with a 5% ZnO concentration in ABS; however, further improvement of the material formulation is needed. As an example, a combination of the two sizes of ZnO, or even of doped ZnO or a pure and doped TiO_2_ semiconductor, could be employed so that the respective composite materials could totally prevent biofilm accumulation with reduced environmental toxicity risks. 

## Figures and Tables

**Table 1:** ABS and ZnO contents in the two types of 3D-printed composite materials.

Composition of Nanosized ZnO Containing Samples		Composition of Microsized ZnO Containing Samples	
ABS	ZnO Nano	ABS	ZnO Micro
80%	20%	80%	20%
90%	10%	90%	10%
95%	5%	95%	5%
97.5%	2.5%	97.5%	2.5%
99%	1%	99%	1%

**Table 2:** Mean crystallite values calculated using Scherrer’s equation before and after *Navicula* and *Chlorella* exposure.

Sample	REF, τ (nm)	Navicula, τ (nm)	Chlorella, τ (nm)
0.5% ZnO micro	31.8	30.0	26.1
2.5% ZnO micro	29.5	24.4	25.0
5% ZnO micro	31.5	25.4	24.7
20% ZnO micro	32.1	23.5	26.1
0.5% ZnO nano	32.3	26.8	21.0
2.5% ZnO nano	26.5	24.8	24.3
5% ZnO nano	28.6	27.8	23.8
20% ZnO nano	28.6	26.5	24.5

## Data Availability

The raw and processed data required to reproduce these findings cannot be shared at this time due to technical or time limitations. The raw and processed data will be provided upon reasonable request to anyone interested anytime until the technical problems will be solved.
